# Supplementary material for: Security of quantum key distribution from generalised entropy accumulation
Source: Nat Commun. 2023 Aug 29;14:5272. doi: 10.1038/s41467-023-40920-8 (PMC10465525; doi:10.1038/s41467-023-40920-8)
Supplement: Supplementary file 1 — Supplementary Information [file 41467_2023_40920_MOESM1_ESM.pdf]

SUPPLEMENTARY NOTES

A. REDUCTION TO ENTROPIC CONDITION

In this section, we provide the detailed proof that Claim 10 implies Theorem 4 (from the main text) using the same ideas as Ref. [1, Section 4.2]. We continue with the same notation as in the main text. As a first step, we add to ρ additional systems C^n defined by

$$C_i = \textsc{ev}(V_i, I_i, S_i)\,. \tag{1}$$

This means that the system C_i is generated the same way as $\hat{C}_i$, except that we use Alice's actual raw key S_i instead of Bob's guess $\hat{S}_i$. We now define the following events (formally defined as subsets of possible values of the classical systems $S^n, \hat{S}^n, \hat{C}^n, C^n$, and E'):

Ω_g: $S^n = \hat{S}^n$ (i.e. Bob's guess of Alice's raw key is correct).

Ω_{KV}: $\textsc{Hash}(S^n) = \textsc{Hash}(\hat{S}^n)$ (i.e. the raw key validation step (Step (5)) passes).

$\Omega_{\hat{C}}$: $\textsc{ca}(\mathsf{freq}(\hat{C}^n)) \geq k_{\text{CA}}$ (i.e. the statistical check (Step (6)) passes using the values $\hat{C}^n$).

Ω_C: $\textsc{ca}(\mathsf{freq}(C^n)) \geq k_{\text{CA}}$ (i.e. the statistical check (Step (6)) passes using the values C^n).

The event Ω of the protocol not aborting is $\Omega = \Omega_{\text{KV}} \wedge \Omega_{\hat{C}}$. If $S^n = \hat{S}^n$, then $\textsc{Hash}(S^n) = \textsc{Hash}(\hat{S}^n)$ and $C^n = \hat{C}^n$. Therefore,

$$\Omega \wedge \Omega_g = \Omega_{\text{KV}} \wedge \Omega_{\hat{C}} \wedge \Omega_g = \Omega_C \wedge \Omega_g\,. \tag{2}$$

Since Step (5) employs a universal hash function, the probability that the protocol does not abort despite $S^n \neq \hat{S}^n$ is at most ε_{KV}, i.e. $\Pr\left[\Omega_g^c \wedge \Omega\right] \leq \varepsilon_{\text{KV}}$, where Ω_g^c is the complement of Ω_g. Hence, we can bound the l.h.s. of Equation (45) by

$$\left\|\rho_{KI^nE'_nE'\wedge\Omega} - \tau_K \otimes \rho_{I^nE'_nE'\wedge\Omega}\right\|_1 \tag{3}$$

$$\leq \left\|\rho_{KI^nE'_nE'\wedge\Omega\wedge\Omega_g} - \tau_K \otimes \rho_{I^nE'_nE'\wedge\Omega\wedge\Omega_g}\right\|_1 + \left\|\rho_{KI^nE'_nE'\wedge\Omega\wedge\Omega_g^c}\right\|_1 + \left\|\tau_K \otimes \rho_{I^nE'_nE'\wedge\Omega\wedge\Omega_g^c}\right\|_1 \tag{4}$$

$$\leq \left\|\rho_{KI^nE'_nE'\wedge\Omega_C\wedge\Omega_g} - \tau_K \otimes \rho_{I^nE'_nE'\wedge\Omega_C\wedge\Omega_g}\right\|_1 + 2\,\varepsilon_{\text{KV}}\,. \tag{5}$$

For the remainder of the proof, we will assume that

$$\Pr[\Omega_g|\Omega_C] \geq \varepsilon_s \text{ and } \Pr[\Omega_C] \geq \varepsilon_a\,. \tag{6}$$

This assumption is justified by the fact that otherwise, we have

$$\Pr[\Omega_C \wedge\ \Omega_g] \leq \min\{\Pr[\Omega_g|\Omega_C]\,, \Pr[\Omega_C]\} \leq \max\{\varepsilon_s, \varepsilon_a\}\,, \tag{7}$$

in which case the theorem statement follows directly from Supplementary Eq. (5) and

$$\left\|\rho_{KI^nE'_nE'\wedge\Omega_C\wedge\Omega_g} - \tau_K \otimes \rho_{I^nE'_nE'\wedge\Omega_C\wedge\Omega_g}\right\|_1 \leq \left\|\rho_{KI^nE'_nE'\wedge\Omega_C\wedge\Omega_g}\right\|_1 + \left\|\tau_K \otimes \rho_{I^nE'_nE'\wedge\Omega_C\wedge\Omega_g}\right\|_1 \tag{8}$$

$$= 2\Pr[\Omega_C \wedge\ \Omega_g]\,. \tag{9}$$

Defining the state $\rho_{KI^nE'_nE'(|\Omega_C)(\wedge\Omega_g)} = 1/\Pr[\Omega_C]\,\rho_{KI^nE'_nE'\wedge\Omega_C\wedge\Omega_g}$, clearly

$$\left\|\rho_{KI^nE'_nE'\wedge\Omega_C\wedge\Omega_g} - \tau_K \otimes \rho_{I^nE'_nE'\wedge\Omega_C\wedge\Omega_g}\right\|_1 \leq \left\|\rho_{KI^nE'_nE'(|\Omega_C)(\wedge\Omega_g)} - \tau_K \otimes \rho_{I^nE'_nE'(|\Omega_C)(\wedge\Omega_g)}\right\|_1\,. \tag{10}$$

Therefore, to show the theorem, it suffices to show that

$$\left\|\rho_{KI^nE'_nE'(|\Omega_C)(\wedge\Omega_g)} - \tau_K \otimes \rho_{I^nE'_nE'(|\Omega_C)(\wedge\Omega_g)}\right\|_1 \leq \varepsilon_{\text{PA}} + 4\,\varepsilon_s\,. \tag{11}$$

The state $\rho_{KI^nE'_nE'(|\Omega_C)(\wedge\Omega_g)}$ is produced by applying a strong extractor in Step (7). Comparing Definition 6 and Supplementary Eq. (11) and remembering that the seed μ is chosen uniformly at random by Alice and is part of the system E', we see that we need to show that

$$H_{\min}^{\varepsilon_s}(S^n|I^nE'_nE')_{\rho_{KE'_nE'(|\Omega_C)(\wedge\Omega_g)}} \geq l + \lceil 2\log(1/\varepsilon_{\text{PA}})\rceil\,. \tag{12}$$

To this end, we can first bound the l.h.s. by

$$H^{\varepsilon_s}_{\min}(S^n|I^nE'_nE')_{\rho_{(|\Omega_C)(\wedge\Omega_g)}} \geq H^{\varepsilon_s}_{\min}(S^n|I^nE'_nE')_{\rho_{|\Omega_C}} \tag{13}$$

$$\geq H^{\varepsilon_s}_{\min}(S^n|I^nC^nE'_nE')_{\rho_{|\Omega_C}} \tag{14}$$

$$\geq H^{\varepsilon_s}_{\min}(S^n|I^nC^nE'_n)_{\rho_{|\Omega_C}} - \lambda_{\text{EC}} - \lceil \log(1/\varepsilon_{\text{KV}}) \rceil\,, \tag{15}$$

where the first inequality follows from [2, Lemma 10] together with the first condition in Supplementary Eq. (6), the second inequality holds because conditioning on additional classical information C^n can only decrease the min-entropy, and the third inequality is a chain rule for the min-entropy which uses the fact that the only information in E' that is correlated with S^n is the error correction information $\text{EC} \in \{0,1\}^{\lambda_{\text{EC}}}$ and the hash $\textsc{Hash}(S^n) \in \{0,1\}^{\lceil \log(1/\varepsilon_{\text{KV}}) \rceil}$. We can show a lower bound on $H^{\varepsilon_s}_{\min}(S^n|I^nC^nE)_{\rho_{|\Omega_C}}$ using the GEAT. Since this is the core of the security proof, we present it as a separate claim. From the preceding discussion and the assumption $\Pr[\Omega_C] \geq \varepsilon_a$ it is then clear that Claim 10 implies Theorem 4.

B. COMPLETENESS

To prove the completeness of the protocol in Box I, we need to bound the probability of the protocol aborting for a given choice of parameters and noise model. Throughout this section, for a fixed choice of arguments in Box I and a fixed noise model $\mathcal{N}$, we denote by ν^{hon} the corresponding "honest single-round state", i.e. formally the state ν^{hon} from Definition 2 when one chooses Eve's collective attack as $\mathcal{N}$. Furthermore, we assume that in Step (4) Alice and Bob use the one-way error correction protocol from [3], which is essentially optimal. We note that in Box I the choice of error correction protocol has no effect on the security statement, only on the completeness statement, so one can also use a heuristic protocol with some fixed leakage λ_{EC} leading to a heuristic value of $\varepsilon^{\text{comp}}_{\text{KV}}$ without impacting the security of the protocol.

There are two steps in which the protocol in Box I may abort: Step (5) if $\textsc{Hash}(S^n) \neq \textsc{Hash}(\hat{S}^n)$, and Step (6) if $\text{CA}(\mathsf{freq}(\hat{C}^n)) < k_{\text{CA}}$. Since $S^n = \hat{S}^n$ implies $\textsc{Hash}(S^n) = \textsc{Hash}(\hat{S}^n)$, we can bound the total abort probability by $\Pr\left[S^n \neq \hat{S}^n\right] + \Pr\left[S^n = \hat{S}^n \wedge \text{CA}(\mathsf{freq}(\hat{C}^n)) < k_{\text{CA}}\right]$. We denote these probabilities by $\varepsilon^{\text{comp}}_{\text{KV}}$ and $\varepsilon^{\text{comp}}_{\text{EV}}$, respectively, so the total abort probability is bounded by $\varepsilon^{\text{comp}}_{\text{KV}} + \varepsilon^{\text{comp}}_{\text{EV}}$.

Supplementary Lemma 1. Fix a noise model $\mathcal{N}$ and a choice of arguments in Box I. Then, any desired value of $\varepsilon^{\text{comp}}_{\text{KV}}$ can be achieved as long as the following condition holds:

$$\lambda_{\text{EC}} \geq nH(S|VI)_{\nu^{\text{hon}}} + 2\sqrt{n}\sqrt{1 - 2\log(\varepsilon^{\text{comp}}_{\text{KV}}/2)}\,\log(1 + 2|\mathcal{S}|) + 2\log\frac{2}{\varepsilon^{\text{comp}}_{\text{KV}}}\,. \tag{16}$$

Proof. By [3], it suffices to show that

$$\lambda_{\text{EC}} \geq H^{\tilde{\varepsilon}}_{\max}(S^n|V^nI^n)_{(\nu^{\text{hon}})^{\otimes n}} + 2\log\frac{1}{\varepsilon^{\text{comp}}_{\text{KV}} - \tilde{\varepsilon}}\,, \tag{17}$$

where $\tilde{\varepsilon} \in [0, \varepsilon^{\text{comp}}_{\text{KV}})$ is a parameter that can be optimised over. For simplicity, here we choose $\tilde{\varepsilon} = \varepsilon^{\text{comp}}_{\text{KV}}/2$, but note that one could numerically optimise over $\tilde{\varepsilon}$ if one wishes to derive the best possible completeness error. By the $H_{\max}$-version of [4, Corollary 4.10],

$$H^{\varepsilon^{\text{comp}}_{\text{KV}}/2}_{\max}(S^n|V^nI^n) \leq nH(S|VI)_{\nu^{\text{hon}}} + 2\sqrt{n}\sqrt{1 - 2\log(\varepsilon^{\text{comp}}_{\text{KV}}/2)}\,\log(1 + 2|\mathcal{S}|)\,. \tag{18}$$

Combining these two equations yields the lemma.

Supplementary Lemma 2. Fix a noise model $\mathcal{N}$ and a choice of arguments in Box I. Then, any desired value of $\varepsilon^{\text{comp}}_{\text{EV}}$ can be achieved as long as the following condition holds for $\delta = \text{CA}(\nu^{\text{hon}}_C) - k_{\text{CA}}$:

$$\delta^2 \geq \frac{2(\mathsf{Max}(\text{CA}) - \mathsf{Min}(\text{CA}))\delta + 6\mathsf{Var}(\text{CA})}{3n}\log\frac{1}{\varepsilon^{\text{comp}}_{\text{EV}}}\,. \tag{19}$$

Proof. Using the definitions at the start of Supplementary Note A, we can write $\varepsilon^{\text{comp}}_{\text{EV}} = \Pr\left[\Omega_g \wedge \Omega^c_{\hat{C}}\right]$. Since $\Omega_g \wedge \Omega^c_{\hat{C}} = \Omega_g \wedge \Omega^c_C$, we can bound $\varepsilon^{\text{comp}}_{\text{EV}} \leq \Pr[\Omega^c_C]$. The honest implementation is i.i.d., so the state we need to

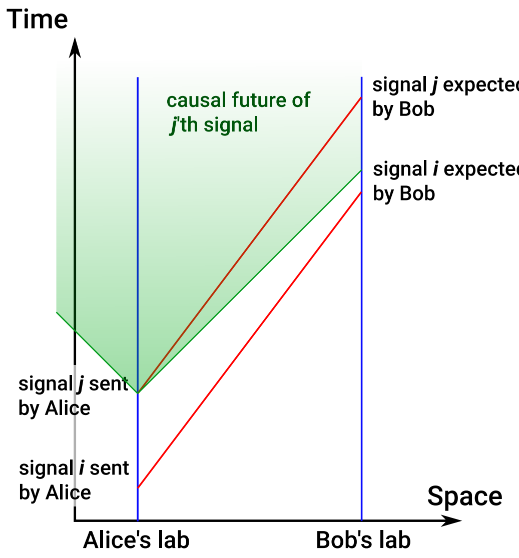

Supplementary Figure 1: Spacetime diagram illustrating causal structure of signal transmission. If Alice and Bob send the signals on a pre-agreed schedule, the spacetime points where Alice sends signals and Bob expects signals are fixed. The causal future (green shaded region) of signal j contains all spacetime points to which Eve can transmit information about signal j. If Eve were not able to speed up the signals at all, then the boundary of the causal future (green line) would coincide with Alice's and Bob's expected signal transmission speed (red line) and the sequentiality assumption would be ensured already between subsequent signals (i.e. Condition 1). If Eve can speed up the signals as shown in the figure, Alice and Bob can choose i and j sufficiently far apart (i.e. choose a sufficiently large step size in Supplementary Condition 11) that the expected arrival of signal i by Bob lies outside the causal future of signal j sent by Alice.

consider in Step (6) is $(\nu^{\text{hon}})^{\otimes n}$. Let $C_1, \dots, C_n$ be i.i.d. random variables with distribution ν_C^{hon}. Then we can view the value k computed by Bob in Step (6) as a random variable, too, and because $\textsc{ca}$ is affine we have

$$k = \frac{1}{n} \sum_{i=1}^{n} \textsc{ca}(\delta_{C_i})\,, \qquad \textsc{ca}(\nu_C^{\text{hon}}) = \mathbb{E}\, \textsc{ca}(\delta_{C_i})\,, \tag{20}$$

where by $\textsc{ca}(\delta_{C_i})$ we mean the random variable that maps a value $c \in \mathcal{C}$ of C_i to $\textsc{ca}(\delta_c)$, with δ_c the point distribution with all the weight on element c. Conditioned on $S^n = \hat{S}^n$, Step (6) aborts if $k < k_{\textsc{ca}}$. We can now apply Bernstein's inequality and find that

$$\Pr\left[\textsc{ca}(\nu_C^{\text{hon}}) - k > \delta\right] \leq \exp\left(-n \frac{\delta^2/2}{\text{Var}[\textsc{ca}(\delta_{C_i})] + (\mathsf{Max}(\textsc{ca}) - \mathsf{Min}(\textsc{ca}))\delta/3} \right) . \tag{21}$$

Requiring that this be less than $\varepsilon_{\text{EV}}^{\text{comp}}$ and noting that by Equation (36), $\text{Var}[\textsc{ca}(\delta_{C_i})] \leq \mathsf{Var}(\textsc{ca})$, we find the desired result.

We note that for simple protocols, e.g. ones where a certain number of rounds are used as "test rounds" that can either pass or fail, the above bound can usually be replaced by a simpler and tighter one using Hoeffding's inequality (see e.g. [5, Section 3.2]).

C. RELAXING THE SEQUENTIALITY ASSUMPTION

As explained in Results Subsection "Modelling Eve's attack", for QKD implementations that allow the adversary Eve to speed up up the transmission of signals, it may be difficult to enforce Condition 1 without significantly lowering the frequency with which signals are sent from Alice to Bob. It is therefore useful to relax Condition 1 and allow Eve to be in possession of s signals at a time. More formally, using the same notation as in Results Subsection "Modelling Eve's attack", we would like to prove security of a prepare-and-measure protocol under the following weaker condition:

Supplementary Condition 11. Eve can only be in possession of at most s subsequent systems $Q_i, \dots, Q_{i+s-1}$ at the same time. We call s the step size of Eve's attack.

We note that this condition does not mean that Eve has to process the signals in disjoint blocks of size s; instead, this condition allows Eve to e.g. first apply an attack on systems $Q_1, \dots, Q_s$, then send system Q_1 to Bob and receive Q_{s+1} from Alice, apply an attack to $Q_2, \dots, Q_{s+1}$, etc. In other words, Eve can execute a "rolling attack" that always uses s adjacent signals. As explained in Results Subsection "Modelling Eve's attack", this condition can be enforced by Alice and Bob using a pre-agreed schedule on which to send their signals, assuming we can place some bound on the amount by which Eve could speed up the transmission of signals from Alice to Bob. (The trivial bound is of course always that Eve can speed up the signal transmission to the speed of light.) We emphasise that to enforce Supplementary Condition 11 (for some appropriately chosen s), Alice and Bob do not need to lower the frequency with which they send signals or divide their signals into blocks with breaks between blocks.

We can now prove an analogous statement to our main result Theorem 4 that only requires the weaker Supplementary Condition 11 instead of Condition 1. The cost that we have to pay for allowing the weaker condition Supplementary Condition 11 is that the second-order term $\frac{g(\varepsilon_s)+\alpha\log(1/\varepsilon_a)}{\alpha-1}$ from Theorem 4 now acquires a prefactor s (and, less importantly, ε_s gets replaced by $\varepsilon_s/(3s-2)$) and we get an additional term $(s-1)g(\varepsilon_s/(3s-2))$; the latter is negligible compared to the former because α is close to 1. However, the first-order term remains unchanged and is independent of s, so in particular the asymptotic key rate (against general attacks with any fixed step size) is the same as in Theorem 4. We illustrate this for the example of B92 in Supplementary Figure 3.

Supplementary Theorem 1. Fix any choice of arguments n, ψ_{UQ}, $\{N^{(v)}\}_{v\in\mathcal{V}}$, PD, RK, EV, k_{CA}, λ_{EC}, ε_{KV}, and ε_{PA} for Box I. Let $\text{CA}:\mathbb{P}(\mathcal{C})\to\mathbb{R}$ be an affine collective attack bound for this choice of arguments. For any $\varepsilon_s, \varepsilon_a > 0, \alpha\in(1,3/2)$, and $s\in\mathbb{N}$, choose a final key length l that satisfies

$$l \le n\,k_{\text{CA}} - n\,\frac{\alpha-1}{2-\alpha}\,\frac{\ln(2)}{2}V^2 - s\,\frac{g(\varepsilon')+\alpha\log(1/\varepsilon_a)}{\alpha-1} - n\left(\frac{\alpha-1}{2-\alpha}\right)^2 K'(\alpha) - (s-1)g(\varepsilon') - \lceil 2\log(1/\varepsilon_{\text{PA}})\rceil - \lceil\log(1/\varepsilon_{\text{KV}})\rceil - \lambda_{\text{EC}}\,, \quad (22)$$

where $g(\cdot)$, V, and $K'(\cdot)$ are defined in Theorem 9 and $\varepsilon' := \frac{\varepsilon_s}{3s-2}$. With this choice of parameters and assuming that Supplementary Condition 11 holds for the value of s chosen above, the protocol in Box I is ε^{cor}-correct and ε^{sec}-secret for

$$\varepsilon^{\text{cor}} = \varepsilon_{\text{KV}}\,, \qquad \varepsilon^{\text{sec}} = \max\{\varepsilon_{\text{PA}} + 4\,\varepsilon_s, 2\,\varepsilon_a\} + 2\,\varepsilon_{\text{KV}}\,. \quad (23)$$

The proof of Supplementary Theorem 1 follows the same steps as the proof of Theorem 4 in Methods Subsection "Proof of main theorem", except that we will need to make some modifications to account for the more general structure of the attack. Since most of the proof is identical, we only provide a sketch and point out the main differences compared to Methods Subsection "Proof of main theorem".

As in Methods Subsection "Proof of main theorem", we again denote the final state at the end of the protocol in Box I (for any fixed attack of the form above) by

$$\rho_{U^nV^nI^nS^n\hat{S}^nC^nK\hat{K}E'_nE'}\,. \quad (24)$$

The system labels here are as in Methods Subsection "Proof of main theorem". The reduction from Theorem 4 to Claim 10 did not use the structure of the attack, so the same steps also allow us to reduce Supplementary Theorem 1 to the following claim.

Supplementary Claim 12. Let Ω_C be the event that $\text{CA}(\mathsf{freq}(C^n)) \ge k_{\text{CA}}$ (i.e. the statistical check (Step (6)) passes using the values C^n). Then, for any $\alpha\in(1,3/2)$:

$$H_{\min}^{\varepsilon_s}(S^n|I^nC^nE'_n)_{\rho_{|\Omega_C}} \ge n\,k_{\text{CA}} - n\,\frac{\alpha-1}{2-\alpha}\,\frac{\ln(2)}{2}V^2 - s\,\frac{g(\varepsilon')+\alpha\log(1/\Pr[\Omega_C])}{\alpha-1} - n\left(\frac{\alpha-1}{2-\alpha}\right)^2 K'(\alpha) - (s-1)g(\varepsilon')\,, \quad (25)$$

with $g(\cdot)$, V, and $K'(\cdot)$ as in Theorem 9 and $\varepsilon' := \frac{\varepsilon_s}{3s-2}$.

Under Supplementary Condition 11, we can model Eve's attacks by a sequence of maps $\mathcal{A}_i : E'_{i-1}Q_i^{i+s-1} \to E'_iQ_i^{i+s-1}$; the difference to the scenario in Results Subsection "Modelling Eve's attack" is that now Eve can act on all

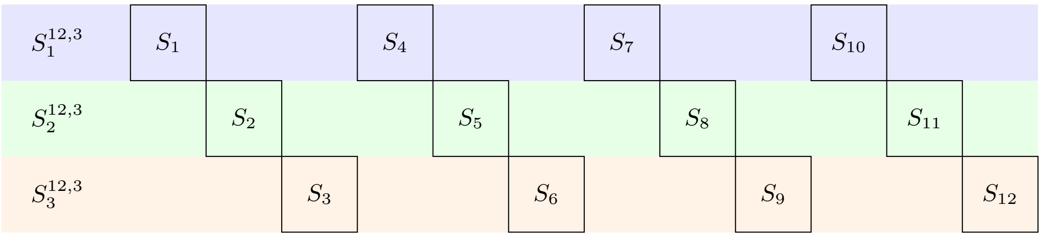

Supplementary Figure 2: Example of how the rounds are split up into interleaved groups for $n = 12$ and $s = 3$.

of $Q_i^{i+s} = Q_i \cdots Q_{i+s-1}$ at the same time. This prevents us from writing the final state ρ as the output of a sequence of maps $\mathcal{M}_i$ as in Claim 10.

To circumvent this issue, we will need to split the systems S^n into interleaved groups of systems

$$S_i^{n,s} \coloneqq S_i S_{i+s} S_{i+2s} \cdots S_{i+\lfloor n/s \rfloor s} \,. \tag{26}$$

This is illustrated in Supplementary Figure 2. We can bound the entropy of S^n in terms of a sum of entropies of the individual groups $S_i^{n,s}$ for $i = 1, \ldots, s-1$ by repeatedly applying the chain rule for min-entropies [6] for a total of $(s-1)$ number of times. Setting $\varepsilon' = \frac{\varepsilon_s}{3s-2}$ as in Supplementary Claim 12, we get that

$$H_{\min}^{\varepsilon_s}(S^n | I^n C^n E'_n)_{\rho_{|\Omega_C}} \tag{27}$$
$$= H_{\min}^{\varepsilon_s}(S_1^{n,s} \ldots S_s^{n,s} | I^n C^n E'_n)_{\rho_{|\Omega_C}} \tag{28}$$
$$\geq H_{\min}^{\varepsilon_s - 3\varepsilon'}(S_1^{n,s} \ldots S_{s-1}^{n,s} | I^n C^n E'_n)_{\rho_{|\Omega_C}} + H_{\min}^{\varepsilon'}(S_s^{n,s} | I^n C^n E'_n S_1^{n,s} \ldots S_{s-1}^{n,s})_{\rho_{|\Omega_C}} - g(\varepsilon') \tag{29}$$
$$\begin{aligned} &\geq H_{\min}^{\varepsilon_s - 2\cdot 3\varepsilon'}(S_1^{n,s} \ldots S_{s-2}^{n,s} | I^n C^n E'_n)_{\rho_{|\Omega_C}} \\ &\qquad + H_{\min}^{\varepsilon'}(S_{s-1}^{n,s} | I^n C^n E'_n S_1^{n,s} \ldots S_{s-2}^{n,s})_{\rho_{|\Omega_C}} + H_{\min}^{\varepsilon'}(S_s^{n,s} | I^n C^n E'_n S_1^{n,s} \ldots S_{s-1}^{n,s})_{\rho_{|\Omega_C}} - 2 \cdot g(\varepsilon') \end{aligned} \tag{30}$$
$$\geq \ldots \tag{31}$$
$$\geq H_{\min}^{\varepsilon_s - (s-1)\cdot 3\varepsilon'}(S_1^{n,s} | I^n C^n E'_n)_{\rho_{|\Omega_C}} + \sum_{i=2}^{s} H_{\min}^{\varepsilon'}(S_i^{n,s} | I^n C^n E'_n S_1^{n,s} \ldots S_{i-1}^{n,s})_{\rho_{|\Omega_C}} - (s-1) \cdot g(\varepsilon') \tag{32}$$
$$= \sum_{i=1}^{s} H_{\min}^{\varepsilon'}(S_i^{n,s} | I^n C^n E'_n S_1^{n,s} \ldots S_{i-1}^{n,s})_{\rho_{|\Omega_C}} - (s-1) \cdot g(\varepsilon') \,, \tag{33}$$

where the last line holds because $\varepsilon_s - (s-1) \cdot 3\varepsilon' = \varepsilon'$. Having split the total entropy into such groups, we can now use the GEAT to bound each $H_{\min}^{\varepsilon'}(S_i^{n,s} | I^n C^n E'_n S_1^{n,s} \ldots S_{i-1}^{n,s})_{\rho_{|\Omega_C}}$ in terms of single-round von Neumann entropies. This works in exactly the same manner as the proof of Claim 10 because by assumption, Eve cannot act simultaneously on more than one of the rounds corresponding to $S_i^{n,s} \coloneqq S_i S_{i+s} S_{i+2s} \cdots S_{i+\lfloor n/s \rfloor s}$. Therefore, for each individual group $S_i^{n,s}$, Eve's attack is a sequential attack on that group of rounds. As a result, following the same steps as in the proof of Claim 10 (and assuming that n is divisible by s for simplicity) we get that for each group of rounds,

$$H_{\min}^{\varepsilon'}(S_i^{n,s} | I^n C^n E'_n S_1^{n,s} \ldots S_{i-1}^{n,s})_{\rho_{|\Omega_C}} \geq \frac{n}{s} \, k_{\text{CA}} - \frac{n}{s} \frac{\alpha - 1}{2 - \alpha} \frac{\ln(2)}{2} V^2 - \frac{g(\varepsilon') + \alpha \log(1/\Pr[\Omega_C])}{\alpha - 1} - \frac{n}{s} \left(\frac{\alpha - 1}{2 - \alpha} \right)^2 K'(\alpha) \,. \tag{34}$$

Note that the extra conditioning on $S_1^{n,s} \ldots S_{i-1}^{n,s}$ does not make a difference to the proof as we may formally consider these systems as part of Eve's side information for that particular group of rounds. Inserting this bound into Supplementary Eq. (33), we get that

$$H_{\min}^{\varepsilon_s}(S^n | I^n C^n E'_n)_{\rho_{|\Omega_C}} \geq n \, k_{\text{CA}} - n \frac{\alpha - 1}{2 - \alpha} \frac{\ln(2)}{2} V^2 - s \, \frac{g(\varepsilon') + \alpha \log(1/\Pr[\Omega_C])}{\alpha - 1} - n \left(\frac{\alpha - 1}{2 - \alpha} \right)^2 K'(\alpha) - (s-1) g(\varepsilon') \tag{35}$$

as claimed in Supplementary Claim 12.

Finally, we note that for certain parameter regimes one can improve the second-order terms in Supplementary Theorem 1 using exactly the same idea as above, but performing the splitting into interleaved groups of rounds at the

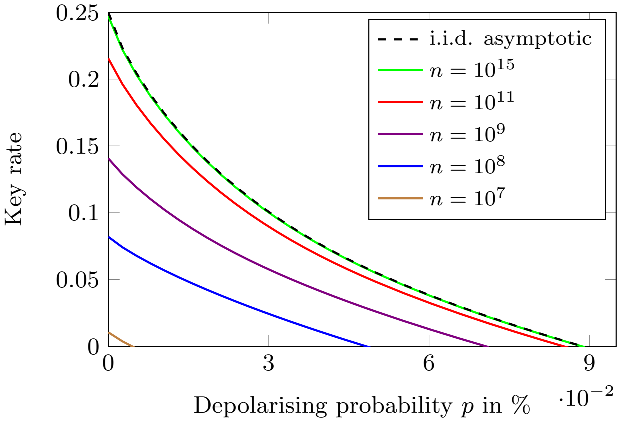

(a) Step size $s = 10$.

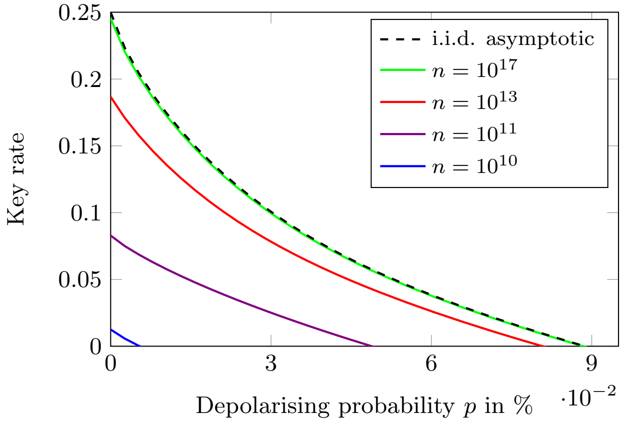

(b) Step size $s = 10^4$. Note that the same colors here do not correspond to the same n as in (a).

Supplementary Figure 3: B92 example with weakened sequentiality condition. We consider the same B92 protocol with the same parameters as in the main text (Results Subsection "Sample application: B92 protocol", see in particular Figure 1), except that we weaken the sequentiality condition to allow for some step size s, and as a result have to use Supplementary Theorem 1 to obtain the key rates. The step size $s = 10$ (Panel (a)) is realistic for satellite-to-earth QKD experiments [9]: let us consider the worst case, where Eve is able to speed up the signals to the speed of light in vaccuum. This defines the largest possible causal future in Supplementary Figure 1 that is still compatible with special relativity. Using the effective thickness of the atmosphere $d \approx 8$km [10] and letting $n_{\text{Air}} \approx 1.0003$ be the refractive index of air, if the signal is sent at a 45 degree angle through the atmosphere, the delay of the signal compared to one travelling at the speed of light c is $\Delta t = \sqrt{2}(n_{\text{Air}} - 1)d/c \approx 10^{-8}s$. Assuming a signal frequency $f_{\text{Signal}} = 1$ GHz (which exceeds the one used e.g. by [9]), we see that a step size $s = \Delta t \cdot f_{\text{Signal}} \approx 10$ is sufficient. In contrast, the plot for $s = 10^4$ (Panel (b)) illustrates what happens at larger block sizes that are more relevant for fibre-based QKD implementations. We see that while the asymptotic key rate remains the same irrespective of block size, at larger block sizes finite-size corrections become more relevant.

level of Renyi entropies, not min-entropies. Concretely, this means that instead of deriving Supplementary Eq. (33), one first relates $H_{\min}^{\varepsilon_s}(S^n|I^nC^nE'_n)_{\rho_{|\Omega_C}}$ to the Renyi entropy (see [7, Definition 5.2]) $H_\alpha^\uparrow(S^n|I^nC^nE'_n)_{\rho_{|\Omega_C}}$, and then applies the chain rule from [8] according to a binary tree of depth $O(\log s)$, i.e. on the first application of the chain rule one splits the rounds into two equally sized groups (corresponding to a step size of 2), on the second application one again splits each of these groups into equally sized subgroups (corresponding to a step size of 4), and so on, until after $O(\log s)$ repetitions the desired step size s is reached. The remainder of the analysis is then identical.

D. ENTANGLEMENT-BASED PROTOCOLS

Our general entanglement-based QKD protocol is very similar to the prepare-and-measure protocol in the Results Section. The only difference is in the the data generation step: in Box I, Alice prepared a state ψ_{UQ} and Bob measured system Q, storing his outcome in register V. In contrast, in Supplementary Box I, Eve prepares a state ψ_{PQE}, and sends P to Alice and Q to Bob. Then, Alice and Bob measure their respective systems, recording the outcomes in registers U and V. The raw data in U and V is then treated exactly the same as in Box I. Even though Step (2)-Step (7) are identical to Box I, we spell out the full protocol for reference.

The rest of this section proceeds similarly to the Results Section: we first explain how to model Eve's attack, again distinguishing between general and collective attacks. We formally define collective attack bounds for the protocol in Supplementary Box I in Supplementary Definition 13. Then, in Supplementary Theorem 2, we analyse the security of the protocol in Supplementary Box I assuming a collective attack bound. The definitions and the security proof are very similar to the Results Section, so we give less detailed explanations and only point out the relevant differences for the proof.

Eve's attack in the protocol in Supplementary Box I is specified by her choice of the state $\psi_{P^nQ^nE}$. An honest Eve would distribute some desired product state $\hat{\psi}$, e.g. an EPR pair, and keep no side information, i.e. $\psi_{P^nQ^nE} = \hat{\psi}_{PQ}^{\otimes n}$. The most general attack available to Eve consists in preparing an arbitrary state $\psi_{P^nQ^nE}$. We note that Eve's attack

Protocol arguments

$n \in \mathbb{N}$:	number of rounds
$\{M^{(u)}\}_{u\in\mathcal{U}}$, $\{N^{(v)}\}_{v\in\mathcal{V}}$:	POVMs acting on Hilbert spaces $\mathcal{H}_P, \mathcal{H}_Q$, respectively, describing Alice's and Bob's measurements with $\mathcal{U}$ and $\mathcal{V}$ the set of possible outcomes
$\text{PD} : \mathcal{U} \times \mathcal{V} \to \mathcal{I}$:	function describing transcript of public discussion (where $\mathcal{I}$ is some finite alphabet)
$\text{RK} : \mathcal{U} \times \mathcal{I} \to \mathcal{S}$:	function describing Alice's raw key generation (where $\mathcal{S}$ is the alphabet of the raw key)
$\text{EV} : \mathcal{V} \times \mathcal{I} \times \mathcal{S} \to \mathcal{C}$:	function "evaluating" each round by assigning a label from the alphabet $\mathcal{C}$
$k_{\text{CA}} > 0$:	required amount of single-round entropy generation
$\varepsilon_{\text{KV}}, \varepsilon_{\text{PA}} > 0$:	tolerated errors during key validation and privacy amplification steps
$\text{CA} : \mathbb{P}(\mathcal{C}) \to \mathbb{R}$:	function corresponding to collective attack bound
$l \in \mathbb{N}$:	length of final key

Protocol steps

(1) *Data generation.* Alice receives systems P^n and Bob systems Q^n of an initial quantum state $\psi_{P^nQ^nE}$ prepared by Eve. For each $i \in \{1, \ldots, n\}$, Alice measures the POVM $\{M^{(u)}\}_{u\in\mathcal{U}}$ on register P_i of the state $\psi_{P^nQ^nE}$ and records the outcome in register U_i. Similarly, Bob measures $\{N^{(v)}\}_{v\in\mathcal{V}}$ on register Q_i and records the outcome in register V_i.

(2) *Public discussion.* For each $i \in \{1, \ldots, n\}$, Alice and Bob publicly exchange information $I_i = \text{PD}(U_i, V_i)$.

(3) *Raw key generation.* For each $i \in \{1, \ldots, n\}$, Alice computes $S_i = \text{RK}(U_i, I_i)$.

(4) *Error correction.* Alice and Bob publicly exchange information $\text{EC} \in \{0,1\}^{\lambda_{\text{EC}}}$, which can depend on U^n, V^n, and I^n. Bob computes $\hat{S}^n(\text{EC}, V^n, I^n) \in \mathcal{S}^n$.

(5) *Raw key validation.* Alice chooses a function $\text{HASH} : \mathcal{S}^n \to \{0,1\}^{\lceil \log(1/\varepsilon_{\text{KV}}) \rceil}$ from a universal hash family $\mathcal{F}$ (Definition 5) according to the associated probability distribution $P_{\mathcal{F}}$ and publishes a description of f and the value $\text{HASH}(S^n)$. Bob computes $\text{HASH}(\hat{S}^n)$ and aborts the protocol if $\text{HASH}(S^n) \neq \text{HASH}(\hat{S}^n)$.

(6) *Statistical check.* For each $i \in \{1, \ldots, n\}$, Bob sets $\hat{C}_i = \text{EV}(V_i, I_i, \hat{S}_i)$. Bob then computes $\text{CA}(\mathsf{freq}(C^n))$. If the result is less than k_{CA}, he aborts the protocol.

(7) *Privacy amplification.* Alice and Bob convert their registers S^n and $\hat{S}^n$ to a binary representation, obtaining strings of length m. Alice chooses a seed $\mu \in \{0,1\}^m$ uniformly at random and publishes her choice. Alice and Bob compute l-bit strings $K = \text{EXT}(S^n, \mu)$ and $\hat{K} = \text{EXT}(\hat{S}^n, \mu)$, respectively, and $\text{EXT} : \{0,1\}^m \times \{0,1\}^m \to \{0,1\}^l$ is a quantum-proof strong $(l + \lceil 2\log(1/\varepsilon_{\text{PA}}) \rceil, \varepsilon_{\text{PA}})$-extractor (Definition 6).

Supplementary Box I: General entanglement-based QKD protocol

in an entanglement-based protocol is not subject to a sequentiality condition (Condition 1) as in a prepare-and-measure protocol. This is because in an entanglement-based protocol, Eve's attack occurs at the level of the input state, which can be arbitrary, and the actions in the protocol performed by Alice and Bob are sequential irrespective of Eve's choice of input state. In contrast, in a prepare-and-measure protocol, Eve's attack is part of the actions performed during the protocol and therefore needs to be modelled as part of the quantum channels applied during the protocol; as a result, for the protocol as a whole to still have a sequential structure, Eve's attack needs to have such a structure, too. This is not an artefact of the GEAT, but rather a structural difference between entanglement-based and prepare-and-measure protocols.

As in the Results Section, a collective attack is the special case where Eve behaves in an i.i.d. manner, i.e. Eve prepares a product state $\psi_{P^nQ^nE} = \psi_{PQE}^{\otimes n}$ for some arbitrary state ψ_{PQE}. Formally, we can define a collective attack bound for Supplementary Box I similarly to Definition 2.

Supplementary Definition 13 (Collective attack bound for Supplementary Box I). Fix arguments $\{M^{(u)}\}_{u\in\mathcal{U}}$, $\{N^{(v)}\}_{v\in\mathcal{V}}$, PD, RK, and EV for Supplementary Box I. Suppose that Alice and Bob run a single round (i.e. $n = 1$) of the protocol in Supplementary Box I up to (and including) Step (3). For a choice of Eve's state ψ_{PQE}, denote the state at the end of Step (3) as ν_{UVSIE}. Let ν_{UVSIEC} be an extension of this state, where $C = \text{EV}(V, I, S)$. A collective attack bound (for the choice of parameters fixed above) is a map $\text{CA} : \mathbb{P}(\mathcal{C}) \to \mathbb{R}$ such that for any initial state ψ_{PQE} prepared by Eve, the state ν_{CUVSIE} satisfies

$$H(S|IEC)_\rho \geq \text{CA}(\rho_C)\,. \tag{36}$$

It is easy to see that for states that minimize the l.h.s. of this inequality, the system E is a purification of P and Q. Hence, it suffices to restrict our attention to such states. We are now ready to prove the security statement for the protocol in Supplementary Box I.

Supplementary Theorem 2. Fix any choice of arguments n, $\{M^{(u)}\}_{u\in\mathcal{U}}$, $\{N^{(v)}\}_{v\in\mathcal{V}}$, PD, RK, EV, k_{CA}, ε_{KV}, and ε_{PA} for Supplementary Box I. Let $\text{CA} : \mathcal{C} \to \mathbb{R}$ be an affine collective attack bound for this choice of arguments. For any $\varepsilon_s, \varepsilon_a > 0$ and $\alpha \in (1, 3/2)$, choose a final key length l that satisfies

$$l \le n\, k_{\text{CA}} - n\,\frac{\alpha-1}{2-\alpha}\,\frac{\ln(2)}{2} V^2 - \frac{g(\varepsilon_s) + \alpha\log(1/\varepsilon_a)}{\alpha-1} - n\left(\frac{\alpha-1}{2-\alpha}\right)^2 K'(\alpha) - \lceil 2\log(1/\varepsilon_{\text{PA}})\rceil - \lambda_{\text{EC}} - \lceil \log(1/\varepsilon_{\text{KV}})\rceil\,,$$

where

$$g(\varepsilon_s) = \log(1-\sqrt{1-{\varepsilon_s}^2})\,, \tag{37}$$

$$V = \log(2|\mathcal{S}|^2+1) + \sqrt{2+\mathsf{Var}(\text{CA})}\,, \tag{38}$$

$$K'(\alpha) = \frac{(2-\alpha)^3}{6(3-2\,\alpha)^3\ln 2}\, 2^{\frac{\alpha-1}{2-\alpha}(2\log|\mathcal{S}|+\mathsf{Max}(\text{CA})-\mathsf{Min}_\Sigma(\text{CA}))} \ln^3\left(2^{2\log|\mathcal{S}|+\mathsf{Max}(\text{CA})-\mathsf{Min}_\Sigma(\text{CA})} + e^2\right)\,. \tag{39}$$

With this choice of parameters, the protocol in Supplementary Box I is ε^{cor}-correct and ε^{sec}-secret for

$$\varepsilon^{\text{cor}} = \varepsilon_{\text{KV}}\,, \qquad \varepsilon^{\text{sec}} = \max\{\varepsilon_{\text{PA}} + 4\,\varepsilon_s, 2\varepsilon_a\} + 2\,\varepsilon_{\text{KV}}\,. \tag{40}$$

Proof. The correctness statement is analogous to the proof of Theorem 4, so we focus on the secrecy condition. Alice, Bob, and Eve's joint final state at the end of the protocol is denoted by $\rho_{U^nV^nI^nS^n\hat{S}^n\hat{C}^nK\hat{K}E}$. As in the proof of Theorem 4 add to ρ additional systems C^n defined by

$$C_i = \text{EV}(V_i, I_i, S_i) \tag{41}$$

and define the event Ω_C by the condition $\text{CA}(\mathsf{freq}(C^n)) \ge k_{\text{CA}}$. Then, we can follow the same steps as in the proof of Theorem 4 to reduce the Supplementary Theorem 2 to the following Supplementary Claim 14.

Supplementary Claim 14. Continuing with the notation from before, for any $\alpha \in (1, 3/2)$:

$$H_{\min}^{\varepsilon_s}(S^n|I^nC^nE)_{\rho_{|\Omega_C}} \ge n\,k_{\text{CA}} - n\,\frac{\alpha-1}{2-\alpha}\,\frac{\ln(2)}{2}V^2 - \frac{g(\varepsilon_s)+\alpha\log(1/\Pr_\rho[\Omega_C])}{\alpha-1} - n\left(\frac{\alpha-1}{2-\alpha}\right)^2 K'(\alpha)\,, \tag{42}$$

where $g(\varepsilon_s)$, V, and $K'(\alpha)$ are defined as in Supplementary Theorem 2.

Proof. To make us of the GEAT, we need to write $\rho_{S^nI^nC^nE|\Omega_C}$ as the result of repeatedly applying quantum channels $\mathcal{M}_1, \dots, \mathcal{M}_n$ to Eve's (arbitrary) initial state $\psi_{P^nQ^nE}$ in Supplementary Box I. For this, we define

$$\mathcal{M}_i : P_iQ_i \to S_iI_iC_i \tag{43}$$

as the following channel: given a quantum system $\omega_{P_iQ_i}$,

(i) measure the POVMs $\{M^{(u)}\}_{u\in\mathcal{U}}$ and $\{N^{(v)}\}_{v\in\mathcal{V}}$ on P_i and Q_i respectively, and store the results in registers U_i and V_i,

(ii) set $I_i = \text{PD}(U_i, V_i)$,

(iii) set $S_i = \text{RK}(U_i, I_i)$,

(iv) set $C_i = \text{EV}(V_i, I_i, S_i)$,

(v) trace out registers U_i and V_i.

Comparing the steps of Supplementary Box I and Supplementary Eq. (??) with this definition of $\mathcal{M}_i$, we see that the marginal of ρ on systems $S^nI^nC^nE$ is the same as the output of the maps $\mathcal{M}_i$:

$$\rho_{S^nI^nC^nE} = \mathcal{M}_n \circ \cdots \circ \mathcal{M}_1(\psi_{P^nQ^nE})\,. \tag{44}$$

If we define the systems $E_i = P_{i+1}^n Q_{i+1}^n I^i C^i E$, then by suitable tensoring with the identity map and copying the register C_i we can also view $\mathcal{M}_i$ as a map

$$\tilde{\mathcal{M}}_i : E_{i-1} \to S_i E_i C_i \,. \tag{45}$$

Then, we can also express the final state (which technically now includes two copies of C^n) as

$$\rho_{S^n E_n C^n} = \tilde{\mathcal{M}}_n \circ \cdots \circ \tilde{\mathcal{M}}_1(\psi_{P^n Q^n E}) \,. \tag{46}$$

To apply Theorem 9, we first need to check that the required conditions on the maps $\tilde{\mathcal{M}}_i$ are satisfied. The condition Equation (30) is clearly satisfied as the systems C_i are themselves included in the conditioning system. The no-signalling condition in Theorem 9 is also trivially satisfied in this case since there is no system R_i.

We now want to argue that the collective attack bound $\textsc{ca} : \mathbb{P}(\mathcal{C}) \to \mathbb{R}$ used as an argument in the protocol is a min-tradeoff function for the maps $\{\mathcal{M}_i\}$. By Definition 8, for this we need to show that for any i and any state $\omega^{i-1}_{E_{i-1}\tilde{E}_{i-1}}$ (where $\tilde{E}_{i-1} \equiv E_{i-1}$):

$$\textsc{ca}(\mathcal{M}_i(\omega^{i-1})_{C_i}) \le H(S_i|E_i\tilde{E}_{i-1})_{\tilde{\mathcal{M}}_i(\omega^{i-1})} = H(S_i|I_iC_iP_{i+1}^nQ_{i+1}^nI^{i-1}C^{i-1}E\tilde{E}_{i-1})_{\tilde{\mathcal{M}}_i(\omega^{i-1})} \,. \tag{47}$$

Remembering that $\tilde{\mathcal{M}}_i$ acts as identity on the systems $P_{i+1}^nQ_{i+1}^nI^{i-1}C^{i-1}E\tilde{E}_{i-1}$, we can consider these systems collectively as a purifying system. Since Supplementary Definition 13 allows arbitrary purifying systems, we see that Equation (47) holds for any collective attack bound $\textsc{ca}$ and conclude that $\textsc{ca}$ is a min-tradeoff function for $\{\mathcal{M}_i\}$. By definition, for any $c^n \in \Omega_C$, $\textsc{ca}(\mathsf{freq}(c^n)) \ge k_{\textsc{ca}}$. Therefore, Supplementary Claim 14 follows by applying Theorem 9.

E. SIMPLIFICATION OF THE OPTIMISATION PROBLEM FOR THE B92 PROTOCOL

Here we describe additional simplifications to the numerical optimisation problem from Equation (70) for the case of the B92 protocol as it is specified in Results Subsection "Sample application: B92 protocol". As a first simplification, we exploit the fact that Alice and Bob distinguish between "test rounds", where $T = 1$ and they use the function $\textsc{ev}_{T=1}$, and "data rounds", where $T = 0$ and they perform no statistical check. We can therefore split the state ν as

$$\nu = (1-\gamma)\nu^{(\mathrm{data})} + \gamma\, \nu^{(\mathrm{test})} \,, \tag{48}$$

where $\nu_C^{(\mathrm{data})} = |\bot\rangle\langle\bot|$ and $\nu_C^{(\mathrm{test})}$ is a distribution over $\mathcal{C}' = \{\mathtt{fail}, \mathtt{inc}, \varnothing\}$ determined according to $\textsc{ev}_{T=1}$. We can now apply [11, Lemma V.5], which states the following (translated to our notation): if an affine function $g : \mathbb{P}(\mathcal{C}') \to \mathbb{R}$ satisfies that for any initial state $\hat{\psi}_{PQ}$,

$$g(\nu_C^{(\mathrm{test})}) \le H(S|IEC)_\nu \,, \tag{49}$$

then the affine function $\textsc{ca} : \mathcal{C} \to \mathbb{R}$ for $\mathcal{C} = \mathcal{C}' \cup \{\bot\}$ defined by

$$\textsc{ca}(\delta_c) := \mathsf{Max}(g) + \frac{1}{\gamma}(g(\delta_c) - \mathsf{Max}(g)) \quad \text{for } c \in \mathcal{C}' \tag{50}$$

$$\textsc{ca}(\delta_\bot) := \mathsf{Max}(g) \tag{51}$$

is a collective attack bound. Here, $\mathsf{Max}(g)$ is defined as in Equation (36) and δ_c denotes the point distribution with all weight on element δ_c. To evaluate $\textsc{ca}$ on any distribution ν_C, one simply writes that distribution as a convex combination of such point distributions and uses that $\textsc{ca}$ is affine, i.e. linear under convex combinations. In addition, [11, Lemma V.5] also provides simple formulae for the properties of $\textsc{ca}$ as in Equation (36) in terms of the properties of g. The main advantage of this approach over a direct evaluation of the optimisation problem from Equation (70) is that for small values of γ, the latter often runs into numerical stability issues, whereas the former does not.

The problem of finding a collective attack bound is therefore reduced to finding a function g that satisfies Supplementary Eq. (49). This can be achieved using the same method as in Results Subsecion "Collective attack bounds". We make the ansatz $g(\nu_C^{(\mathrm{test})}) = \vec{\lambda}' \cdot \vec{\nu}_C^{(\mathrm{test})} + c_{\vec{\lambda}'}$ and choose $\vec{\lambda}'$ heuristically, e.g. using Matlab's `fminsearch`. Given a choice of $\vec{\lambda}'$, we need to determine $c_{\vec{\lambda}'}$ such that g satisfies Supplementary Eq. (49). Following the steps of Results Subsecion "Collective attack bounds", we can see that a valid choice of $c_{\vec{\lambda}'}$ is given by the solution to the following convex optimisation problem:

$$c_{\vec{\lambda}'} = \inf_{\hat{\psi}_{PQ}} D\big(\nu^1_{PQSIC} \,\big\|\, \mathcal{P}_S(\nu^1_{PQSIC})\big) - \vec{\lambda}' \cdot \vec{\nu}_C^{(\mathrm{test})} \tag{52}$$

$$\text{s.t. } \hat{\psi}_{PQ} \ge 0 \,, \quad \mathrm{Tr}\Big[\hat{\psi}_{PQ}\Big] = 1 \,, \quad \hat{\psi}_P = \tilde{\psi}_P \,, \tag{53}$$

where ν^1 is as defined in Equation (68). We can simplify this optimisation problem by focussing on the case $I = \top$ and $C = \perp$ since we only expect to generate raw data for the secret key in a data round whose measurement was not inconclusive:

$$D\big(\nu^1_{PQSIC} \,\big\|\, \mathcal{P}_S(\nu^1_{PQSIC})\big) \geq D\big(\nu^1_{PQS\wedge I=\top\wedge C=\perp} \,\big\|\, \mathcal{P}_S(\nu^1_{PQS\wedge I=\top\wedge C=\perp})\big) \,. \tag{54}$$

Note that this inequality always holds by data processing and because I and C is are classical registers, so we always obtain a valid (albeit possibly less tight) key rate after applying it. The preceding explanation argues that in the case of B92, this inequality is (close to) an equality and therefore using it does not significantly lower the key rate.

Computing $M^{(s,i,c)}_{PQ}$ according to Equation (63), inserting this into Equation (68), and using that the square root acts trivially on projectors, we can write $\nu^1_{PQS\wedge I=\top\wedge C=\perp}$ explicitly as

$$\nu^1_{PQS\wedge I=\top\wedge C=\perp} = (1-\gamma) \sum_{s,s'\in\{0,1\}} \Big(|s\rangle\langle s|_P \otimes \sqrt{\mathbb{1} - N_Q^{(\perp)}}\Big) \hat{\psi}_{PQ} \Big(|s'\rangle\langle s'|_P \otimes \sqrt{\mathbb{1} - N_Q^{(\perp)}}\Big) \otimes |s\rangle\langle s'| \,. \tag{55}$$

This state arises from

$$\tilde{\nu}_{PQ\wedge I=\top\wedge C=\perp} = (1-\gamma) \Big(\mathbb{1}_P \otimes \sqrt{\mathbb{1} - N_Q^{(\perp)}}\Big) \hat{\psi}_{PQ} \Big(\mathbb{1}_P \otimes \sqrt{\mathbb{1} - N_Q^{(\perp)}}\Big) \tag{56}$$

by application of the isometry $V = \sum_{s\in\{0,1\}} |s\rangle\langle s|_P \otimes |s\rangle_S$. Similarly we can see that

$$\mathcal{P}_S(\nu^1_{PQS\wedge I=\top\wedge C=\perp}) = V \mathcal{P}_P(\tilde{\nu}_{PQ\wedge I=\top\wedge C=\perp}) V^\dagger \,, \tag{57}$$

so by isometric invariance of the relative entropy:

$$D\big(\nu^1_{PQS\wedge I=\top\wedge C=\perp} \,\big\|\, \mathcal{P}_S(\nu^1_{PQS\wedge I=\top\wedge C=\perp})\big) = D(\tilde{\nu}_{PQ\wedge I=\top\wedge C=\perp} \,\|\, \mathcal{P}_P(\tilde{\nu}_{PQ\wedge I=\top\wedge C=\perp})) \,. \tag{58}$$

Therefore, we can find a valid choice for $c_{\vec{\lambda}}$ by solving the simplified optimisation problem

$$c_{\vec{\lambda}'} = \inf_{\hat{\psi}_{PQ}} D(\tilde{\nu}_{PQ\wedge I=\top\wedge C=\perp} \,\|\, \mathcal{P}_P(\tilde{\nu}_{PQ\wedge I=\top\wedge C=\perp})) - \vec{\lambda}' \cdot \vec{\nu}_C^{(\text{test})} \tag{59}$$

$$\text{s.t. } \hat{\psi}_{PQ} \geq 0 \,, \quad \operatorname{Tr}\Big[\hat{\psi}_{PQ}\Big] = 1 \,, \quad \hat{\psi}_P = \tilde{\psi}_P \,. \tag{60}$$

We solve this optimisation problem using the package `CVXQUAD` [12] and thus obtain the desired affine collective attack bound.

F. PARAMETERS FOR B92 EXAMPLE

In this section, we detail our choice of parameters for the B92 protocol presented in the main text. Most parameter choices are summarised in the following table.

Symbol	Value	Description
n	see Figure 1	number of rounds
ε_{KV}	$5\cdot 10^{-11}$	tolerated error during key validation step
ε_{PA}	10^{-10}	tolerated error during privacy amplification step
ε_a	$4\cdot 10^{-10}$	minimum probability of passing statistical check
ε_s	$2\cdot 10^{-10}$	smoothing parameter
$\varepsilon_{\text{KV}}^{\text{comp}}$	$5\cdot 10^{-3}$	abort probability from key validation step
$\varepsilon_{\text{EV}}^{\text{comp}}$	$5\cdot 10^{-3}$	abort probability from statistical check

We refer to Box I and Theorem 4 for a more detailed description of the role of each parameter. The testing probability γ and the parameter α from Theorem 4 are optimised numerically. We choose the remaining arguments for Box I so that they satisfy the conditions in Supplementary Lemma 1, Supplementary Lemma 2, and Theorem 4 and compute the resulting values of ε^{cor}, ε^{sec}, and $\varepsilon^{\text{comp}}$. Specifically, to choose a value for λ_{EC}, a direct calculation shows that for noise level p, we have $H(S|VI)_{\nu^{\text{hon}}} = \frac{1+p}{4} h(1/(1+p))$, where $h(x) = -x\log x - (1-x)\log(1-x)$ is the

binary entropy, and ν^{hon} is the state for an honest noisy implementation and depends implicitly on the noise level p. We then set

$$\lambda_{\text{EC}} = \left\lceil n\frac{1+p}{4}h(1/(1+p)) + 2\sqrt{n}\sqrt{1-2\log(\varepsilon_{\text{KV}}^{\text{comp}}/2)}\log(7) + 2\log\frac{2}{\varepsilon_{\text{KV}}^{\text{comp}}}\right\rceil . \tag{61}$$

We furthermore choose $k_{\text{CA}} = \text{CA}(\nu_C^{\text{hon}}) - \delta$ for

$$\delta = \left(\frac{2(\mathsf{Max}(\text{CA}) - \mathsf{Min}(\text{CA}))\text{CA}(\nu_C^{\text{hon}}) + 6\mathsf{Var}(\text{CA})}{3n}\log\frac{1}{\varepsilon_{\text{EV}}^{\text{comp}} - \varepsilon_{\text{KV}}}\right)^{1/2} . \tag{62}$$

Note that we have replaced the factor of δ on the r.h.s. in Supplementary Lemma 2 by $\text{CA}(\nu_C^{\text{hon}})$. This can be done since we are only interested in cases where $k_{\text{CA}} > 0$, i.e. we can assume $\delta \leq \text{CA}(\nu_C^{\text{hon}})$. It is easy to see that the above choices satisfy the conditions in Supplementary Lemma 1 and Supplementary Lemma 2, whence it follows that the total completeness error for this choice of parameters is

$$\varepsilon^{\text{comp}} = \varepsilon_{\text{KV}}^{\text{comp}} + \varepsilon_{\text{EV}}^{\text{comp}} = 10^{-2} . \tag{63}$$

Finally, we choose the key length to be the largest integer l that satisfies the condition in Equation (12). Then, we can apply Theorem 4 to find that the B92 protocol with this choice of parameters is ε^{cor}-correct and ε^{sec}-secret with

$$\varepsilon^{\text{cor}} = \varepsilon_{\text{KV}} = 5\cdot 10^{-11} , \qquad \varepsilon^{\text{sec}} = \max\{\varepsilon_{\text{PA}} + 4\,\varepsilon_s, 2\,\varepsilon_a\} + 2\,\varepsilon_{\text{KV}} \leq 10^{-9} . \tag{64}$$

G. BB84 PROTOCOL WITH DECOY STATES

The BB84 protocol [13] is the most well-known QKD protocol and we already described how it can be viewed as an instance of Box I. That description assumed that Alice always sends a single qubit to Bob. This qubit could e.g. be implemented as a polarised photon. However, in practice Alice will usually use a highly attenuated laser that does not reliably output a single photon. Instead, the number s of photons in a laser pulse is distributed according to the Possonian distribution $p_L(s|\mu) = e^{-\mu}\frac{\mu^s}{s!}$, where the average photon number μ depends on the laser's intensity and is known to Alice. This means that a single pulse may contain multiple photons (with the same polarisation), allowing Eve to perform a photon number splitting attack [14]: Eve measures the number of photons in a pulse and, if there are multiple photons, measures the polarisation of one of the photons and forwards the others to Bob unchanged; this tells Eve the corresponding bit in the raw key. Therefore, only raw key bits from rounds with exactly one photon in the laser pulse contribute to the secret key. We therefore need to lower-bound the fraction Ω of "single-photon rounds" and the error rate e_1 in those rounds. While such a bound can be obtained from the standard BB84 protocol, the resulting key rate is quite poor. The decoy state method is a modification of the BB84 protocol that allows for better estimation of Ω and e_1 and therefore achieves higher key rates; see [15–18] for a background on the idea of decoy state protocols.

Decoy-state protocols can be implemented with commercially available components and thus serve as good examples for practical QKD. The purpose of this section is to show that the methods developed here can be used to establish their security. For this, we give a formal description of the BB84 decoy state protocol as an instance of Box I and explain how an existing i.i.d. asymptotic analysis of the BB84 decoy state protocol can be understood as a collective attack bound in our framework. This puts us in a position to apply Theorem 4: we simply need to compute the relevant properties of the collective attack bound and numerically optimize over the parameters of the protocol to obtain the maximum key rate. As our goal is to illustrate the use of our framework, and not to numerically optimize key rates, we leave a detailed numerical analysis with experimentally realistic noise models for future work. This example again highlights the ease of use of our framework: one only needs to verify that the protocol fits into the template Box I and can reuse collective attack bounds from prior work to immediately obtain a finite-size key rate against general attacks. In contrast to previous techniques such as the de Finetti theorem, using photonic protocols over qubit protocols introduces no additional complications because our Theorem 4 only depends on the collective attack bound, not the underlying quantum states.

We now describe the BB84 decoy state protocol as an instance of Box I. We note that there are many different variants of the decoy state BB84 protocol. Here, we use the version described in [18], except that we use a fixed number of total rounds. We denote by $\psi_{\mu,x,a}$ the state produced by a laser pulse at intensity μ with (polarisation) basis $x \in \{\mathsf{Z}, \mathsf{X}\}$ and value (polarisation direction) $a \in \{0,1\}$. In the protocol, Alice chooses an intensity $\mu \in \{\mu_1, \mu_2, \mu_3\}$ with probabilities $p_{\mu_1}, p_{\mu_2}, p_{\mu_3}$, respectively, for some μ_i satisfying $\mu_1 > \mu_2 + \mu_3$ and $\mu_2 > \mu_3 \geq 0$. Alice also chooses

a basis $x \in \{\mathsf{Z}, \mathsf{X}\}$ with probability q_x, and a value $a \in \{0,1\}$ uniformly at random. Therefore,

$$\psi_{UQ} = \sum_{\substack{\mu \in \{\mu_1,\mu_2,\mu_3\} \\ x \in \{\mathsf{Z},\mathsf{X}\}}} p_\mu q_x |\mu, x, a\rangle\langle \mu, x, a|_U \otimes (\psi_{\mu,x,a})_Q \,. \tag{65}$$

Bob will choose a measurement basis $y \in \{\mathsf{Z}, \mathsf{X}\}$ with probability $q_y = q_x$ and measure the signal he receives from Alice. The measurement will yield an outcome $b \in \{\emptyset, 0, 1\}$, where $\emptyset$ corresponds to Bob not detecting any photon and $0, 1$ denote Bob's measured polarisations. This measurement can be described by a POVM $\{N^{(y,b)}\}$. Because we will reuse a collective attack bound from [18] instead of deriving our own, there is no need to write out this POVM explicitly.

During public discussion, Alice announces the intensity μ, Bob announces whether he received outcome $\emptyset$ or not, and both reveal their basis choices. Furthermore, if $x = y = \mathsf{Z}$, they announce their values a and b. Formally, denoting by $U_i = (\mu_i, x_i, a_i)$ and $V_i = (y_i, b_i)$ Alice's and Bob's classical values in the i-th round,

$$\textsc{pd}(U_i, V_i) = \begin{cases} (\mu_i, x_i, y_i, a_i, b_i) & \text{if } x_i = y_i = \mathsf{Z} \wedge b_i \neq \emptyset \\ (\mu_i, x_i, y_i, \emptyset) & \text{if } b_i = \emptyset \\ (x_i, y_i) & \text{else.} \end{cases} \tag{66}$$

Alice uses the measurement outcomes from rounds where both her and Bob chose the X-basis as the raw key (with $I_i = \textsc{pd}(U_i, V_i)$):

$$S_i = \textsc{rk}(U_i, I_i) = \begin{cases} a_i & \text{if } x_i = y_i = \mathsf{X}\,, \\ \perp & \text{else.} \end{cases} \tag{67}$$

Finally, to evaluate each round, Bob records the intensity, whether he received outcome $\emptyset$ when Alice sent a photon in the X-basis, and whether their outcomes agree in case $x_i = y_i = \mathsf{Z}$. Formally,

$$\textsc{ev}(V_i, I_i, \hat{S}_i) = \begin{cases} (\mu_i, \emptyset_{\mathsf{X}}) & \text{if } x_i = \mathsf{X} \wedge\, b_i = \emptyset, \\ (\mu_i, \mathtt{err}_{\mathsf{Z}}) & \text{if } x_i = y_i = \mathsf{Z} \wedge\, a_i \neq b_i \neq \emptyset, \\ \perp & \text{else.} \end{cases} \tag{68}$$

We therefore see that decoy state protocols naturally fit into the framework of Box I.

The decoy state BB84 protocol is simple enough to be analysed analytically in the i.i.d. asymptotic setting. Therefore, instead of using the numerical technique from Results Subsecion "Collective attack bounds", we can instead reuse these analytical results as our collective attack bound. Concretely, we need to bound $H(S|IEC)_\nu$ for any collective attack. For the decoy state BB84 protocol, this analysis can be performed analytically, so we do not need to invoke the numerical technique described in the main text. We briefly sketch the derivation of the analytical bound and refer to [17, 18] for details.

We define the "transmission probability" t_s^x as the probability that if Alice sends out an s-photon state in basis $x \in \{\mathsf{Z}, \mathsf{X}\}$, Bob will detect at least one photon, i.e. not receive outcome $\emptyset$. Note that $t_0^x > 0$, i.e. Bob may detect a photon even though Alice did not send one, either due to a dark count in Bob's detector or due to Eve sending a photon instead. Similarly, the "failure probability" f_s^x is defined as the probability that if Alice sends out s photons in basis x, Bob's measurement outcome b will be different from Alice's chosen value a, conditioned on Bob not receiving $\emptyset$. We note that because Alice only knows the intensity of her laser, not how many photons are in a particular pulse, the above quantities are not directly accessible to Alice and Bob. Also recall that Alice chooses intensity $\mu \in \{\mu_1, \mu_2, \mu_3\}$ with probability p_{μ_i}, and that for a given choice of μ the number of photons is distributed as

$$p_L(s|\mu) = e^{-\mu} \frac{\mu^s}{s!} \,. \tag{69}$$

The security analysis now proceeds in two steps: first, we bound $H(S|IEC)_\nu$ in terms of t_s^x and f_s^x. Then we bound the latter quantities in terms of statistics Alice and Bob can observe in the protocol. To bound $H(S|IEC)_\nu$, we observe that if Alice did not send any photons but Bob had a detection, the corresponding raw key bit has 1 bit of entropy. On the other hand, if Alice sent exactly 1 photon, the standard BB84 analysis based on uncertainty relations [19] applies and the corresponding raw key bit has $1 - h(f_1^{\mathsf{Z}})$ bits of entropy, where h is the binary entropy function. Finally, if Alice sends multiple photons, the corresponding raw key bit cannot be guaranteed to have any

entropy due to the photon number splitting attack. Since we consider the photon number to be a classical quantity, we can simply take the weighted average over these cases and find that for any collective attack,

$$H(S|IEC)_\nu \geq \tau_0 t_0^{\mathsf{X}} + \tau_1 t_1^{\mathsf{X}}(1 - h(f_1^{\mathsf{Z}}))\,, \tag{70}$$

where τ_s is the probability that Alice sends out and s-photon state:

$$\tau_s = \sum_{i=1}^{3} p_{\mu_i} p_L(s|\mu_i)\,. \tag{71}$$

To bound t_s^x and f_s^x, we define the corresponding quantities t_μ^x and f_μ^x as the transmission and failure probabilities if Alice sends out a laser pulse with intensity μ. The t_μ^x and f_μ^x are observable quantities because Alice knows which intensity she set for her laser. Clearly,

$$t_{\mu_i}^x = \sum_{s=0}^{\infty} p_L(\mu_i|s) t_s^x\,, \text{ and } f_{\mu_i}^x = \sum_{s=0}^{\infty} p_L(\mu_i|s) f_s^x\,, \tag{72}$$

where

$$p_L(\mu_i|s) = \frac{p_{\mu_i}}{\tau_s} p_L(s|\mu_i) \tag{73}$$

according to Bayes' rule. From this, one can derive the following bounds by straightforward algebra (see [17, 18] for details):

$$t_0^x \geq \frac{\mu_2 - \mu_3}{\tau_0} \left(\frac{\mu_2 e^{\mu_3} t_{\mu_3}^x}{p_{\mu_3}} - \frac{\mu_3 e^{\mu_2} t_{\mu_2}^x}{p_{\mu_2}} \right), \tag{74}$$

$$t_1^x \geq \frac{\mu_1 \tau_1}{\mu_1(\mu_2 - \mu_3) - (\mu_2^2 - \mu_3^2)} \left(\frac{e^{\mu_2} t_{\mu_2}^x}{p_{\mu_2}} - \frac{e^{\mu_3} t_{\mu_3}^x}{p_{\mu_3}} + \frac{\mu_2^2 - \mu_3^2}{\mu_1^2} \left(\frac{t_0^x}{\tau_0} - \frac{e^{\mu_1} t_{\mu_1}^x}{p_{\mu_1}} \right) \right), \tag{75}$$

$$f_1^x \leq \frac{\tau_1}{\mu_2 - \mu_3} \left(\frac{e^{\mu_2} f_{\mu_2}^x}{p_{\mu_2}} - \frac{e^{\mu_3} f_{\mu_3}^x}{p_{\mu_3}} \right). \tag{76}$$

We now note that $t_{\mu_i}^{\mathsf{X}}$ and $f_{\mu_i}^{\mathsf{Z}}$ are related to the statistics stored in register C by

$$t_{\mu_i}^{\mathsf{X}} = 1 - \Pr[C = (\mu_i, \emptyset_{\mathsf{X}})]\,, \quad f_{\mu_i}^{\mathsf{Z}} = \Pr[C = (\mu_i, \texttt{err}_{\mathsf{Z}})]\,. \tag{77}$$

Therefore, if we insert Supplementary Eq. (76) into Supplementary Eq. (70), we obtain a collective attack bound $\textsc{ca}(\nu_C)$. To use Theorem 4, one can take any affine lower bound to this function.

---

[1] E. Y.-Z. Tan, P. Sekatski, J.-D. Bancal, R. Schwonnek, R. Renner, N. Sangouard, and C. C.-W. Lim, Improved DIQKD protocols with finite-size analysis, arXiv preprint arXiv:2012.08714 (2020).
[2] M. Tomamichel and A. Leverrier, A largely self-contained and complete security proof for quantum key distribution, Quantum **1**, 14 (2017).
[3] J. M. Renes and R. Renner, One-shot classical data compression with quantum side information and the distillation of common randomness or secret keys, IEEE Transactions on Information Theory **58**, 1985 (2012).
[4] F. Dupuis, O. Fawzi, and R. Renner, Entropy accumulation, Communications in Mathematical Physics **379**, 867 (2020).
[5] R. Arnon-Friedman, R. Renner, and T. Vidick, Simple and tight device-independent security proofs, SIAM Journal on Computing **48**, 181 (2019).
[6] A. Vitanov, F. Dupuis, M. Tomamichel, and R. Renner, Chain rules for smooth min-and max-entropies, IEEE Transactions on Information Theory **59**, 2603 (2013).
[7] M. Tomamichel, *Quantum information processing with finite resources: mathematical foundations*, Vol. 5 (Springer, 2015).
[8] F. Dupuis, Chain rules for quantum rényi entropies, Journal of Mathematical Physics **56**, 022203 (2015).
[9] S.-K. Liao, W.-Q. Cai, W.-Y. Liu, L. Zhang, Y. Li, J.-G. Ren, J. Yin, Q. Shen, Y. Cao, Z.-P. Li, *et al.*, Satellite-to-ground quantum key distribution, Nature **549**, 43 (2017).
[10] Glossary of meteorology: homogeneous atmosphere, American Meteorological Society.
[11] F. Dupuis and O. Fawzi, Entropy accumulation with improved second-order term, IEEE Transactions on information theory **65**, 7596 (2019).

[12] H. Fawzi, J. Saunderson, and P. A. Parrilo, Semidefinite approximations of the matrix logarithm, Foundations of Computational Mathematics **19**, 259 (2019), package cvxquad at https://github.com/hfawzi/cvxquad.

[13] C. H. Bennett and G. Brassard, Quantum cryptography: Public key distribution and coin tossing, in *Proceedings of IEEE International Conference on Computers, Systems and Signal Processing* (1984) pp. 8, vol. 175.

[14] N. Lütkenhaus and M. Jahma, Quantum key distribution with realistic states: photon-number statistics in the photon-number splitting attack, New Journal of Physics **4**, 44 (2002).

[15] W.-Y. Hwang, Quantum key distribution with high loss: toward global secure communication, Physical review letters **91**, 057901 (2003).

[16] H.-K. Lo, X. Ma, and K. Chen, Decoy state quantum key distribution, Physical review letters **94**, 230504 (2005).

[17] X. Ma, B. Qi, Y. Zhao, and H.-K. Lo, Practical decoy state for quantum key distribution, Physical Review A **72**, 012326 (2005).

[18] C. C. W. Lim, M. Curty, N. Walenta, F. Xu, and H. Zbinden, Concise security bounds for practical decoy-state quantum key distribution, Physical Review A **89**, 022307 (2014).

[19] M. Berta, M. Christandl, R. Colbeck, J. M. Renes, and R. Renner, The uncertainty principle in the presence of quantum memory, Nature Physics **6**, 659 (2010).
